# Aggregation of polyQ-extended proteins is promoted by interaction with their natural coiled-coil partners

**DOI:** 10.1002/bies.201300001

**Published:** 2013-03-11

**Authors:** Spyros Petrakis, Martin H Schaefer, Erich E Wanker, Miguel A Andrade-Navarro

**Affiliations:** 1) Neuroproteomics, Max Delbrueck, Center for Molecular MedicineBerlin, Germany; 2) Aristotle University of Thessaloniki, ThessalonikiGreece; 3) Computational Biology and Data Mining, Max Delbrueck Center for Molecular MedicineBerlin, Germany

**Keywords:** coiled-coil, polyglutamine, protein aggregation, protein interactions

## Abstract

Polyglutamine (polyQ) diseases are genetically inherited neurodegenerative disorders. They are caused by mutations that result in polyQ expansions of particular proteins. Mutant proteins form intranuclear aggregates, induce cytotoxicity and cause neuronal cell death. Protein interaction data suggest that polyQ regions modulate interactions between coiled-coil (CC) domains. In the case of the polyQ disease spinocerebellar ataxia type-1 (SCA1), interacting proteins with CC domains further enhance aggregation and toxicity of mutant ataxin-1 (ATXN1). Here, we suggest that CC partners interacting with the polyQ region of a mutant protein, increase its aggregation while partners that interact with a different region reduce the formation of aggregates. Computational analysis of genetic screens revealed that CC-rich proteins are highly enriched among genes that enhance pathogenicity of polyQ proteins, supporting our hypothesis. We therefore suggest that blocking interactions between mutant polyQ proteins and their CC partners might constitute a promising preventive strategy against neurodegeneration.

## Polyglutamine diseases

Polyglutamine (polyQ) diseases are fatal neurological disorders that affect the central nervous system. Nine known disorders belong to this family, which includes Huntington's disease (HD) and spinocerebellar ataxia type-1 (SCA1; see 1 for a review). PolyQ diseases are caused by mutations in disease genes that contain CAG trinucleotide expansions in their coding regions. These mutations are translated into expanded glutamine chains in pathological proteins such as huntingtin (HTT) or ataxin-1 (ATXN1) 2. The threshold for the pathogenic length of polyQ expansions is disease specific and correlates with the age at onset of the disease. For example, in ATXN1, a threshold of approximately 40 glutamines is critical for disease initiation. Usually, the first symptoms manifest around the fourth decade of life, but mutant proteins with 70 or more glutamines can initiate disease before adulthood 3.

Intriguingly, although proteins mutated in polyQ diseases have very different biological functions and are widely expressed in various tissues, their mutant forms cause neurodegenerative diseases, preferentially in specific neuronal cell types. A major question that remains unanswered is why particular neurons are more susceptible to neurodegeneration than others. In the case of SCA1, which is caused by mutant ATXN1 protein 4, a gradual atrophy and loss of cerebellar Purkinje neurons is observed. PolyQ-expanded ATXN1 likely destabilizes its interaction with the partner protein ROR-α 5, which is a known regulator of development and function of Purkinje neurons 6. These data suggest that abnormal interactions between a polyQ-expanded protein and its interacting partners may be responsible for toxicity and cell loss in polyQ diseases 7.

A characteristic pathological hallmark in polyQ diseases is the formation of large inclusion bodies with insoluble protein aggregates in the nuclei of affected neurons. These inclusions consist mainly of polyQ proteins but also contain ubiquitin and components of the protein clearance machinery 8. It is still not clear whether inclusion bodies directly contribute to cytotoxicity or their formation is a cellular response for the storage of toxic polyQ proteins. Experimental evidence suggests that mutant protein monomers or small oligomers rather than large insoluble protein aggregates are the most toxic species in neurodegenerative polyQ diseases 9. Additionally, impairment of the protein clearance machinery due to ageing or oxidative stress 10 and failure to degrade misfolded polyQ proteins cause earlier disease initiation. These observations suggest that aggregates have an important role in the pathogenesis of polyQ diseases.

Because expanded polyQ sequences are preferentially observed in the context of neurodegenerative diseases, polyQ regions have been studied from a pathological point of view with a focus on their possible role in disease. However, polyQ tracts are a feature of more than 60 human proteins 11 and are present in many proteins from different species. Here, we propose that polyQ regions have a significant function that we need to understand, in order to figure out how polyQ expansions result in aggregation and disease.

## Statement of the idea: polyQ tracts as modulators of coiled-coil interactions

PolyQ proteins belong to protein families with many different functions, but a higher preponderance in transcription factors and nuclear proteins has been observed 12. The conservation of polyQ regions and their evolutionary patterns in protein families such as HTT and others suggest that they have an important biological function. Encouraged by this evidence, we recently studied the function of polyQ tracts by association to features in proteins bearing polyQ tracts or interacting with those 13; the stronger correlations we found were the following: (i) proteins with longer polyQ tracts have a higher number of interactors than proteins with short polyQ tracts or without such sequences and (ii) there is a statistically significant presence of predicted coiled-coil (CC) regions next to polyQ regions. A CC domain consists of parallel or antiparallel α-helices that further fold like the strands of a rope. These domains facilitate protein dimerization and are present in proteins with many biological functions 14.

**Figure 1:**
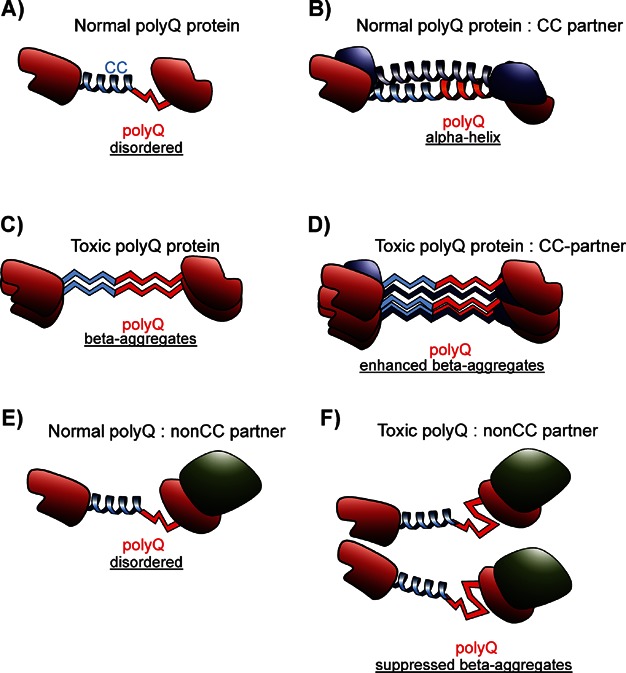
Cartoon models of interactions of polyQ wild-type and mutant proteins with CC and non-CC partners. **A:** A wild-type polyQ protein contains a CC region near a disordered polyQ tract (red). **B:** Upon interaction with a CC partner (blue), the polyQ region adopts a CC conformation and modulates the interaction. **C:** The mutant polyQ protein with an expanded polyQ tract adopts a β-strand conformation and forms β-sheet aggregates. **D:** The CC partner of the wild-type polyQ protein interacts with the aggregates and enhances them. **E:** A non-CC partner of the wild-type polyQ protein (green) binds at a different domain. **F:** This non-CC partner binds to the aggregates of the mutant polyQ protein, sequesters the toxic protein and sterically impedes its aggregation.

Our findings are consistent with the previous suggestion that polyQ modulates protein-protein interactions (PPIs) by expanding a neighboring CC upon interaction with a CC from a partner protein (Fig. 1; 15). Such a CC expansion was observed in X-ray solved structures of the N-terminal fragment of HTT including a helical region, a polyQ and a following polyproline (polyP) domain, fused to the maltose-binding protein 16. We also confirmed a significant occurrence of polyP tracts following a polyQ domain, which might hinder its aggregation by decreasing the probability of the polyQ region to form a β-rich state 17.

A very interesting hypothesis follows immediately from the previous model of polyQ action. It has been shown that proteins with expanded polyQ domains can form β-sheet aggregates 18 (Fig. 1). Such polyQ aggregates form molecular platforms that affect the solubility of protein partners and influence interactions 19. Then, CC proteins that normally interact with the CC-polyQ region might increase aggregation of the polyQ expanded protein (Fig. 1). Therefore, the abnormal interaction between a mutant polyQ protein and its CC-rich partner would stem from the aggregates of the pathological protein and not from its monomers. On the contrary, proteins that interact with the wild-type polyQ protein at a different region (Fig. 1), may sequester the toxic polyQ protein and reduce the formation of aggregates (Fig. 1). These particular effects on the interaction of mutated polyQ proteins with other proteins are complex and may depend on the structure of the interacting proteins. Such hypotheses should be also taken into consideration when investigating the effects of interacting proteins on the toxicity of polyQ expanded proteins.

## Molecular mechanisms of polyQ pathogenesis

We recently performed a cell-based screen for the identification of proteins that influence the aggregation and proteotoxicity of a toxic construct of polyQ ATXN1 (ATXN1Q82, 20). We observed that the set of ATXN1Q82 aggregation enhancers was significantly enriched in proteins with predicted CC domains, while we could not identify predicted CC domains in the set of ATXN1Q82 aggregation suppressors. More specifically, we observed that the protein MED15, which interacts with ATXN1Q82, enhances polyQ protein aggregation through its CC domain. In contrast, Pum1, an ATXN1Q82 partner with no predicted CC conformation, had the opposite effect 20. Since the relation between protein aggregates and the pathology of polyQ diseases is a controversial topic 21, we investigated the cytotoxic effects of ATXN1Q82 aggregation enhancers. We found that while their effects were heterogeneous, indeed most of these aggregation enhancers increased cytotoxicity as well (see Supplementary Table S2 in 20). For example, the CC-rich protein MED15, which induced the formation of intranuclear inclusions, strongly enhanced ATXN1Q82-mediated cytotoxicity as well (20 and unpublished data), indicating that the CC domain in MED15 was critical for both ATXN1Q82 aggregation and cytotoxicity.

The pathology of polyQ diseases may also be triggered by the formation of abnormal protein complexes. It was recently demonstrated that wild-type ATXN1 interacts with both the transcriptional repressor CiC and the splicing factor RBM17 in the nucleus. However, the polyQ-expanded ATXN1 protein showed higher affinity for RBM17 than for CiC, leading to increased amounts of the ATXN1-RBM17 complex compared to the ATXN1-CiC complex 22. Experiments in a SCA1 *Drosophila* model showed that CiC is neuroprotective while RBM17 has the opposite effect 22, 23, suggesting that both CiC and RBM17 directly modulate cytotoxicity through their interaction with polyQ-expanded ATXN1. Following our observations of the relation between CC content and aggregation effects by polyQ interacting proteins, we analyzed the RBM17 and CiC sequences using the COILS program 24. We predicted that RBM17, like the aggregation enhancing protein MED15, contains a large CC-domain at its N-terminus. In contrast, no CC domain was predicted for the cytotoxicity suppressor CiC, which is similar to our observations for the aggregation suppressor Pum1 20. Therefore it seems feasible to speculate that the interaction of polyQ-expanded ATXN1 with CC-rich proteins (such as MED15 or RBM17) might promote disease pathogenesis in SCA1.

Our findings for ATXN1 are affine to our hypothesis that CC partners of polyQ expanded proteins can enhance the aggregation of those proteins. In the next section we explore whether this hypothesis extends to other pathogenic polyQ proteins using data from genetic screens for disease-related polyQ proteins.

## Enhancers of polyQ toxicity/aggregation and CC content

In the previous section we have presented a hypothesis that might explain why enhancers of polyQ-expanded ATXN1 toxicity and aggregation are enriched in CC domains. However, there have been several screens that tested the effect of gene silencing or overexpression on the aggregation and toxicity of polyQ proteins 25–27. Are the obtained results consistent with our hypothesis?

Here we compare an siRNA gene knock-down screen in *Caenorhabditis elegans* to identify modifiers of Q35::YFP aggregation 26, two screens in *Drosophila melanogaster* for modifiers of ataxin-3 27 and HTT 25 toxicity, and our own study of modifiers of YFP-ATXN1Q82^NT^ toxicity mentioned above 20 (Table 1). We use these data to observe a potential enrichment of CC domains in proteins whose overproduction enhanced aggregation or toxicity of polyQ proteins. We investigated the different CC frequencies among these gene sets and compared them to the background frequencies in the respective proteomes. We computed *p* values for all modifier sets using the *χ*^2^-test.

**Table 1:** Enrichment for coiled-coils (CC) in enhancers of polyQ toxicity and aggregation

Refs.	Pathological protein	Organism	Background CC	Test	Enhancers	CC	*p*-Value
20	YFP-ATXN1Q82^NT^	*H. sapiens*	0.17	Toxicity	12	0.50	0.01
27	C-terminal ATXN3Q78	*D. melanogaster*	0.17	Toxicity	49	0.39	9.9e−5
25	N-terminal HTTQ128	*D. melanogaster*	0.17	Toxicity	17	0.35	0.09
26	Q35::YFP	*C. elegans*	0.12	Aggregation	152	0.11	0.69

Background CC, background fraction of predicted CC proteins in the given species; test, toxicity or aggregation; enhancers, number of enhancers reported; CC, fraction of predicted CC enhancers; *p*-value, *p*-value comparing CC frequencies in the enhancers and the background set.

All three studies reporting enhancers of toxicity of real polyQ protein fragments resulted in sets of proteins with a higher content of predicted CC domains than in the corresponding organism background (Table 1). In contrast, the *C. elegans* study, which uses a simple polyQ tract attached to YFP (as opposed to using a polyQ tract and its surrounding CC region), reports a set of aggregation enhancers without enrichment in CC (Table 1).

We conclude that the function of polyQ tracts is to modulate protein interactions in the context of specific surrounding sequences. This is supported by a recent study reporting that the C-terminal region of the RNA-binding protein Nab3 containing a predicted α-helix following a Q16 tract, is sufficient to provide this protein with the ability to self-assemble 28. Obviously, this hypothesis is based on the interpretation of a limited number of results and will need further evidence to be proven or disproven, but it can be taken as a guide for future research. Additional evidence will allow us to better define the interplay between polyQ expansion, interactions with CC-rich partners and protein aggregation.

## Perspectives

Focused studies may provide more insights into the mechanisms of how CC-rich proteins enhance the pathological effects of polyQ proteins. It will be especially important to find out how the transition of an α-helical structure into an aggregation-prone, β-sheet conformation occurs. If CC-rich partner proteins function as aggregation enhancers of polyQ disease proteins, it might be a feasible therapeutic strategy to block these interactions with small molecules or peptides. Hypothetically, molecules with high affinities for polyQ-rich CC domains should prevent the interaction between the polyQ protein and its natural CC partner. At the same time, they might also prevent the conformational conversion of the α-helical structure into the β-sheet structure, which is a prerequisite for the spontaneous formation of insoluble polyQ-containing protein aggregates. Preferably, a CC-mimicking molecule would stabilize the polyQ protein in an α-helical soluble conformation.

In conclusion, several lines of evidence indicate that: (i) CC proteins that interact with a wild-type polyQ protein also interact with the corresponding pathogenic polyQ-extended protein, and (ii) these CC protein partners promote the aggregation of the pathogenic polyQ-extended protein. A careful analysis of these interactions should help us to develop therapeutic strategies that might prevent neurodegeneration in humans. We suggest that similar aggregation mechanisms might be relevant for other non-polyQ neurodegenerative diseases involving protein aggregation, such as Alzheimer's or Parkinson's disease.

## Abbreviations

ATXN1: ataxin-1
CC: coiled-coil;
HD: Huntington's disease
HTT: huntingtin
polyQ: polyglutamine
PPI: protein-protein interaction
SCA1: spinocerebellar ataxia type-1
